# Myofibroblastic CAF Density, Not Activated Stroma Index, Indicates Prognosis after Neoadjuvant Therapy of Pancreatic Carcinoma

**DOI:** 10.3390/cancers14163881

**Published:** 2022-08-11

**Authors:** Ulrike Heger, Anna Martens, Lisa Schillings, Britta Walter, Domenic Hartmann, Ulf Hinz, Thomas Pausch, Nathalia Giese, Christoph W. Michalski, Thilo Hackert

**Affiliations:** 1 Department of General Visceral and Transplantation Surgery, Heidelberg University Hospital, 69120 Heidelberg, Germany; 2 German Cancer Research Center, 69120 Heidelberg, Germany; 3 Dr. Senckenberg Institute of Pathology, Goethe University, 60590 Frankfurt, Germany; 4 Department of General Surgery, University Hospital, 89081 Ulm, Germany

**Keywords:** pancreatic cancer, FOLFIRINOX, paclitaxel, neoadjuvant therapy, tumor stroma, CAF, collagen

## Abstract

**Simple Summary:**

Pancreatic cancer is increasingly treated with chemotherapy before surgery, but this does not improve prognosis for every patient. On a cellular level, pancreatic cancer tissue is usually mixed with a dense matrix called the stroma which interacts with the tumor and contains fibers, large molecules and cells. The role of this stroma in chemotherapy and subsequent surgical tumor resection is unclear, especially for current first-line chemotherapy regimens. We analyzed two of the main stromal components, activated fibroblasts and collagen, and found that treatment with gemcitabine + nab-paclitaxel reduced collagen content while FOLFIRINOX had no quantitative effect. Meanwhile, a higher number of activated fibroblasts was beneficial for prognosis after chemotherapy. These findings will serve to further elucidate mechanisms of response and chemoresistance in pancreatic cancer and potentially to stratify patients for different treatment pathways.

**Abstract:**

Neoadjuvant therapy (NT) for advanced PDAC is an emerging concept, affecting both stroma and tumor. The Activated Stroma Index (ASI; ratio of activated cancer-associated fibroblasts (CAF) to collagen deposition) is a prognostic marker in upfront resected pancreatic adenocarcinoma (PDAC). We assessed ASI and its prognostic relevance after NT. Tissue from resection specimens of *n* = 48 PDAC patients after neoadjuvant chemotherapy with FOLFIRINOX (FOL; *n* = 31), gemcitabine + nab-paclitaxel (GEM; 7) or combination treatment (COMB; 10) was compared with upfront resected matched controls (RES; 69). Activated CAFs were assessed by immunohistochemistry for α-SMA, and collagen was stained with aniline blue; the stained area was then determined by computational imaging analysis and ASI was calculated. In GEM, ASI was significantly higher and collagen deposition lower than in controls and FOL. The lowest quartile of ASI values had significantly longer overall survival (OS) in RES, whereas in FOL, the highest quartile had the best prognosis. After NT, OS was significantly improved in the α-SMA-high group; in RES, however, survival was independent of α-SMA. Reversed prognostic association of ASI thus points to the differing significance of stromal composition after FOL, while improved prognosis with high CAF abundance suggests a synergistic effect of myofibroblasts with chemotherapy. These divergences impede usability of ASI after NT.

## 1. Introduction

Pancreatic ductal adenocarcinoma (PDAC) is a disease with a dismal prognosis, largely due to late diagnosis and lack of screening options as well as relative chemoresistance. Only about 20% of diagnosed patients are in a surgically resectable stage and 5-year overall survival (OS) has seen only slight improvements in the past decades, reaching about 10% [1]. One of the characteristics of PDAC is an extensive desmoplastic reaction consisting of acellular material such as collagen fibers, hyaluronic acid, fibronectin and other matricellular proteins equivalent to the extracellular matrix (ECM), as well as blood vessels, immune cells and activated fibroblasts, together making up a scirrhous mass termed the tumor stroma [2]. An early step in pancreatic ductal carcinogenesis is the activation of fibroblasts and pancreatic stellate cells (PSC) and their transformation by cancer-led signaling via TGF-ß into myofibroblastic cancer-associated fibroblasts (myCAFs) leading to tissue fibrosis by production of large amounts of ECM, among other effects [3,4,5,6]. PSC and stromal fibroblasts are thought to function as regulators of ECM homeostasis, as they secrete protein-degrading enzymes as well, with myCAFs expressing α-smooth muscle antigen (α-SMA) as a specific marker [5,7,8,9,10]. The ECM furthermore produces and stores cytokines such as a variety of growth factors, and this characteristic microenvironment is considered a highly dynamic landscape contributing to chemoresistance [2,4,11]. Erkan et al. demonstrated in upfront resected PDAC that the group of patients with high collagen but low PSC activity had a favorable prognosis [12] and introduced a marker termed activated stroma index (ASI), denoting the ratio of activated PSC to collagen deposition. The complex roles of CAFs and collagen and their regulatory network in PDAC have recently been elucidated, further demonstrating the antitumorigenic effects of myCAFs via collagen I production and detrimental effects after abrogation of collagen production, as well as their direct interaction with immune cells of the stroma [13,14,15].

Gemcitabine monotherapy or radiochemotherapy used to be the first-line chemotherapeutic treatment in PDAC from 1997 [16], until it emerged that the addition of nab-paclitaxel improves survival [17]. This combination therapy has been shown to have a pronounced effect on tumor stroma by depleting it and increasing vascularization, intratumoral gemcitabine concentration and tumor regression. A lot of these data are derived from animal models, though; the few translational studies on stromal effects after gemcitabine/nab-paclitaxel have conflicting results and are generally lacking precise quantification of stromal components [18,19,20].

Since FOLFIRINOX—the combination chemotherapy containing fluorouracil, leucovorin, irinotecan and oxaliplatin—was shown to improve prognosis in palliative and adjuvant treatment of PDAC compared with gemcitabine alone in 2013 [21,22]; it is now considered the gold standard of chemotherapy in PDAC. Thus, FOLFIRINOX has been increasingly used in a neoadjuvant setting for locally advanced, borderline resectable and oligometastatic PDAC, leading to secondary resectability in up to 60% of cases [23]. For patients not tolerating the rather toxic regimen, gemcitabine/nab-paclitaxel is usually seen as noninferior and is approved as first-line therapy in international guidelines [24]. On a tissue level, the treatment effects of FOLFIRINOX are even less understood than those of gemcitabine/nab-paclitaxel. While some of the more recent translational studies contained FOLFIRINOX-treated patients as well, hardly any subgroup analyses were reported. Xie et al. found a higher ASI in 11 samples from FOLFIRINOX-treated patients than after gemcitabine-based therapy, however, without any correlation of ASI with survival [25]. Contrarily, in a recent paper on 31 samples of PDAC after mostly gemcitabine-based neoadjuvant therapy and 13 after FOLFIRINOX, Mota-Reyes et al. found a significantly lower ASI after neoadjuvant treatment [26]; unfortunately, no stratification according to the type of NT is provided, and the cohort contained no nab-paclitaxel-treated patients.

In patients undergoing resection of PDAC after neoadjuvant treatment (NT), oftentimes the tumor mass appears more brittle and, though imaging might still suggest vascular contact, can be removed from visceral arteries by divestment [27]. Hypothesizing an association of this phenomenon with stromal effects of NT, we sought to determine the relevance of ASI values after the current first-line regimens for NT in PDAC and to analyze the effect different types of NT have on collagen deposition and activation of PSC.

## 2. Materials and Methods

### 2.1. Patients

The institutional prospectively maintained database was screened for all patients undergoing surgery for pancreatic cancer between 2013 and 2017 after previous NT with FOLFIRINOX or gemcitabine + nab-paclitaxel. A matched control group was then selected which had undergone upfront resection. Formalin-fixed, paraffin-embedded 3 µm tissue sections of sufficient quality along with complete clinical data were available from 48 NT patients and 69 matched upfront resection patients; demographic and clinicopathological data of patients were complemented retrospectively from electronic patient records. Survival time was calculated from date of diagnosis. Samples were provided by the tissue banks of the National Centre for Tumour Diseases (NCT, Heidelberg, Germany) and the Pancobank of the European Pancreas Centre Heidelberg in accordance with institutional regulations and with the approval of the ethics committee of the University of Heidelberg.

### 2.2. Stainings and Microscopy

**Figure 1:**
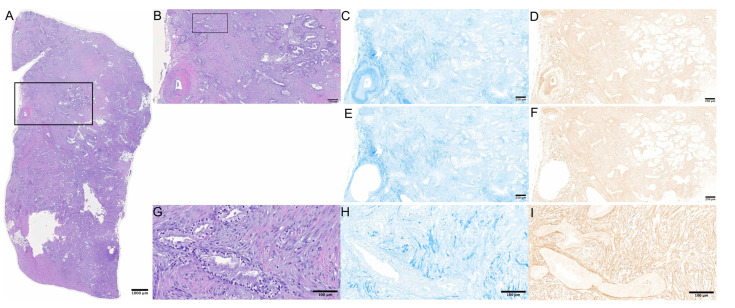
Representative example of histological slides: (**A**) whole slide overview, H&E staining; bar represents 1000 µm. (**B**) Zoom of marked cutout of panel a; bar represents 250 µm. (**C**) Consecutive section corresponding to (**B**), aniline blue staining; (**D**) consecutive section corresponding to (**B**), α-SMA immunohistochemistry; (**E**,**F**) represents (**C**,**D**) after manual exclusion of vessels, respectively; (**G**) zoomed cutout of (**B**); bar represents 100 µm; (**H**) consecutive section corresponding to (**G**), aniline blue staining; (**I**) consecutive section corresponding to (**G**), α-SMA immunohistochemistry.

H&E stainings served for confirmation of PDAC diagnosis and to determine the area of interest. As described by Erkan et al. for analysis of ASI, collagen and α-SMA—originally for detection of activated PSC but recently discovered to be the main marker of the myCAF subtype—were then stained on separate slides [12]. On two consecutive sections each, stainings with α-SMA antibodies (Monoclonal Mouse Anti-Human Smooth Muscle Actin Clone 1A4, DAKO Denmark A/S) and with aniline blue (AB; Anilinblau, C.I. 42780, Carl Roth GmbH + Co. KG) were performed according to instructions of the manufacturer (Representative stainings cf. Figure 1). Slides were then digitalized on the Axio Scan.Z1 (Carl Zeiss Microscopy GmbH) and captured by a Hitachi HV-F202SCL camera (Hitachi Kokusai Electric America, Ltd., Southwick, MA, USA). Individual images taken through a 10x objective were stitched offline, resulting in a pyramid representation. The protocol for tissue analysis followed the techniques described in the original publication by Erkan et al. [12]. Prior to measurements, areas containing smooth muscle cells such as vessels with a diameter of >0.2 µm, duodenal mucosa and fatty tissue were manually excluded from analysis. Afterwards, all images were converted to grayscale (8-bit). Thresholds for further conversion into black and white images were determined by two independent observers (AM and DH). On the final binary digital image, the stained area as equivalent to black area was measured and assessed in percentage of stained total area. ASI was then calculated as follows:

$$\mathrm{ASI}=\frac{\alpha -\mathrm{SMA}\;\mathrm{stained}\;\mathrm{area}\;\left[{\mathrm{mm}}^{2}\right]}{\mathrm{aniline}\;\mathrm{blue}\;\mathrm{stained}\;\left[{\mathrm{mm}}^{2}\right]}$$

### 2.3. Statistical Analysis

All statistical testing was performed with SPSS Statistics software (V.27; IBM Corporation, Armonk, NY, USA). Categorical variables were compared using chi-squared or Fisher′s exact test, depending on cell frequencies. Tissue parameters α-SMA, AB and the resulting ASI were not normally distributed in most subgroups, as assessed by the Shapiro–Wilk test (α = 0.05); thus, nonparametric Mann–Whitney *U* and Kruskal–Wallis tests were used; multiple comparisons were adjusted by Bonferroni correction. Survival times were analyzed by the Kaplan–Meier-method, compared by log-rank test and reported as median with 95% confidence intervals (CI). To analyze survival according to differential tissue marker expression, ASI and its constituents were divided into statistical quartiles or high and low expression (above and below median values, respectively) and the resulting subgroups compared statistically. Two-sided *p*-values of ≤5% were considered statistically significant. Because of the exploratory study design and the descriptive character of the analyses performed, all results were interpreted cautiously, and *p*-values were used descriptively.

## 3. Results

### 3.1. Patient Characteristics

The overall cohort comprised *n* = 117 patients aged 40.0–86.9 years (median: 69.1) of whom *n* = 63 were male (53.8%). Overall, *n* = 69 had undergone upfront resection (RES group; 59.0%) and *n* = 48 had been resected after neoadjuvant treatment (NT group; 41.0%). Of the latter, *n* = 31 had received NT with FOLFIRINOX only (FOL subgroup; 64.6% of NT, median six cycles), *n* = 10 a combination of neoadjuvant treatments (COMB subgroup; 20.8%) and *n* = 7 gemcitabine/nab-paclitaxel only (GEM subgroup; 14.6%, median three cycles). The COMB subgroup included patients who in addition to a median of six cycles of FOLFIRINOX had received partially overlapping gemcitabine/nab-paclitaxel (*n* = 4), radiochemotherapy (*n* = 4), gemcitabine mono (*n* = 3) and single cases with gemcitabine + erlotinib and FOLFOX. In line with our department′s treatment pathways, all patients undergoing NT had either locally advanced or disseminated stages at time of diagnosis, and the majority underwent extended resections. The treatment subgroups differed significantly in age (younger patients in COMB and FOL), ASA classification (lower score in FOL vs. RES) and extent of surgery (higher in FOL than RES; all Table 1). Among the *n* = 23 M1 patients, female sex (*n* = 16; 69.9% of M1) was significantly more frequent than in M0 (*n* = 38 female, 40.4%; *p* = 0.0.012). Other clinicopathological parameters were not statistically different.

### 3.2. Tissue Markers

**Figure 2:**
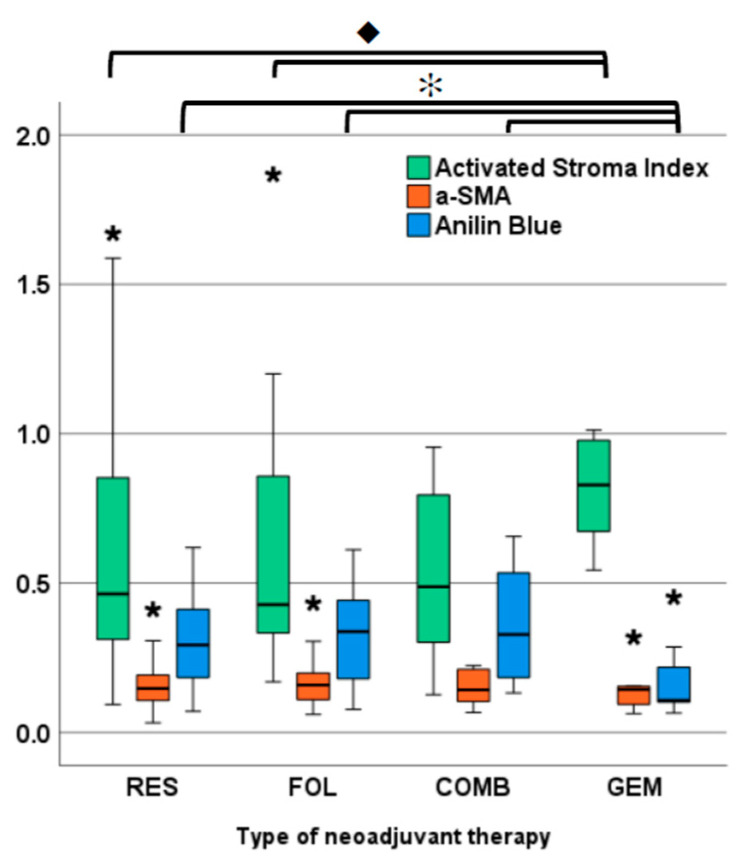
ASI, α-SMA and AB values by type of neoadjuvant therapy. ◆: ASI GEM vs. RES *p* = 0.032; GEM vs. FOL *p* = 0.048. ✻: AB GEM vs. RES *p* = 0.015; GEM vs. FOL *p* = 0.015; GEM vs. COMB *p* = 0.025. Asterisks indicate outlier values (>1.5× IQR and <3× IQR); *n* = 5 extreme outliers clipped from chart (>3× IQR; RES: 3.8/2.6; FOL: 3.9/2.4; GEM: 2.2).

**Figure A1:**
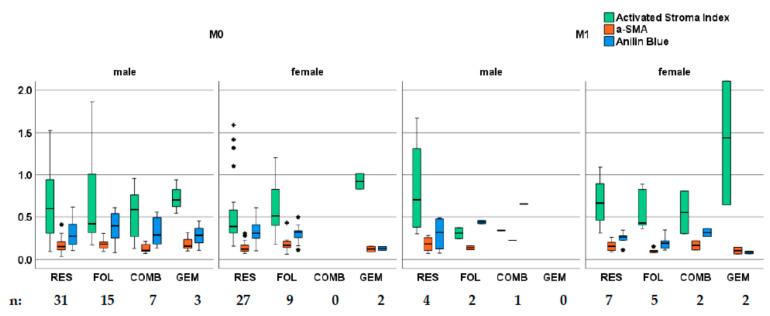
Overview of tissue parameter distribution across subgroups of NT, M status and sex with annotated group sizes. Female patients were more frequent in the M1 subgroup and underrepresented in the COMB subgroup; all male GEM patients were also M0. Asterisks indicate outlier values (>1.5× IQR), + indicates extreme outliers (>3× IQR).

Tissue parameters α-SMA, AB and the resulting ASI were not significantly different between the RES and NT groups (data not shown). In the treatment subgroups, however, ASI showed a distinct trend for higher values in GEM than in all other (sub)groups; this was statistically significant in the Mann–Whitney U tests of GEM vs. both RES and FOL (*p* = 0.032 and 0.048, respectively; Figure 2). Correspondingly, AB values were lower in the GEM group with significant differences compared with RES, FOL and COMB (*p* = 0.015; 0.015; and 0.025) indicating lower collagen content after GEM treatment (Figure 2). After further subgrouping by M status, these trends persisted in both M0 and M1 subgroups, yet lacked statistical significance, likely due to the small sample sizes (Appendix A: Overview of tissue parameter across all subgroups, Figure A1). In contrast, α-SMA values did not exhibit any differences between RES and NT nor between the subgroups (Figure 2).

Comparing tissue parameters between categories of patient characteristics, microscopic venous invasion pV was significantly associated with lower ASI values in the overall cohort (*n* = 45 vs. 36; *p* = 0.045; data not shown) but not in (sub)group analyses. Of note, however, data for lymphatic, venous and perineural invasion were only available in *n* = 81 (69.2%) of all cases. Furthermore, AB values were significantly different between *n* stage categories in the overall cohort, (*p* = 0.021) with N1 significantly higher than N2 (*p* = 0.049) but not N0 (*p* = 0.055). In the NT group, AB values were significantly higher for the small subgroup with perineural invasion than without (*n* = 3 vs. 34; *p* = 0.045).

Additionally, lower AB values were significantly associated with female sex within the NT group (*n* = 20 vs. 28; *p* = 0.010), the M1 group (*n* = 16 female vs. 7 male; *p* = 0.047) and the FOL subgroup (*n* = 14 vs. 17; *p* = 0.032), with a nonsignificant trend in the GEM subgroup as well (*n* = 4 vs. 3). Contrastingly, in the RES group, no significant associations of tissue parameters with sex nor trends were found (Figure A1). In the overall cohort as well as in subgroup analyses for RES/NT, type of NT or M0/M1 stage, no further notable associations were found between tissue parameters and variables shown in Table 1.

### 3.3. Survival

Four patients of the overall cohort had died of other causes than PDAC and were excluded from analysis of overall survival (OS), as were two patients with palliative resections, resulting in a subset of *n* = 111 patients. Tissue parameters of the six excluded patients were not significantly different from the analyzed cohort (data not shown). OS was calculated from date of diagnosis and was not different between RES and NT at a median of 20.3 months (RES; *n* = 66; 95% CI: 17.5–23.1) vs. 24.1 (NT; *n* = 45; 17.8–30.3; *p* = 0.939). M1 patients had a significantly shorter OS at 16.6 months (*n* = 21; 13.6–19.6) than M0 at 23.8 (90; 13.7–33.8; *p* = 0.009). A trend for shorter median OS in female patients was observed within the NT group at a median of 34.4 months for males (*n* = 26; 25.5–43.3) vs. 17.2 for females (*n* = 17; 15.3–19.2; *p* = 0.051). After exclusion of M1 patients due to the imbalanced distribution of sex and M status between types of NT, a significant association of female sex with worse OS was only found within the small M0 GEM subgroup (*n* = 2 vs. 3; 35.0 vs. 12.0 months; *p* = 0.039; data not shown).

### 3.3.1. ASI and Survival

**Figure 3:**
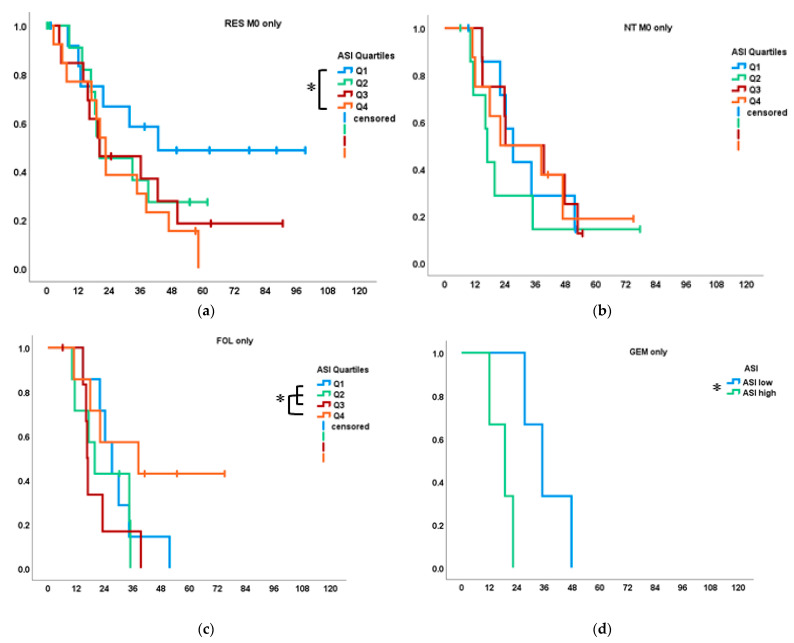

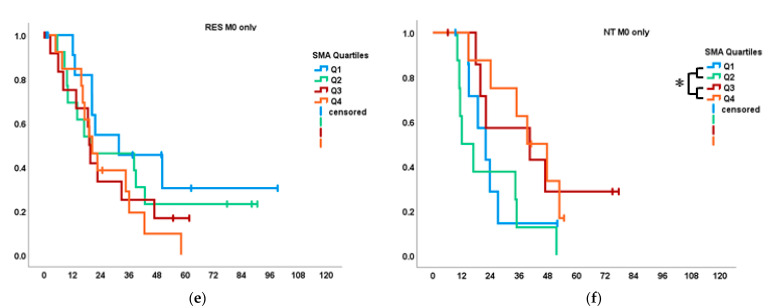
Overall survival (OS; months) of subgroups by tissue parameter quartiles (Q). (**a**) OS of RES M0 according to ASI; each Q *n* = 13 (✻ Q1 vs. Q4: *p* = 0.047); (**b**) OS of NT M0 according to ASI; each Q *n* = 8; (**c**) OS of FOL according to ASI; each Q *n* = 7 (✻ Q4 vs. Q1–3: *p* = 0.045); (**d**) OS of GEM according to ASI; high vs low, each *n* = 3 (*p* = 0.025) (**e**) OS of RES M0 according to α-SMA each Q *n* = 13; (**f**) OS of NT M0 according to α-SMA; each Q *n* = 8 (✻ Q1 + 2 vs. Q3 + 4: *p* = 0.010).

When subdivided into quartiles according to ASI values, the lowest quartile Q1 was associated with significantly longer OS than Q4 (31.7 months (95% CI: 0–67.9) vs. 19.1 (13.2–25.0); *p* = 0.040) in the RES as well as RES M0 group (median OS in Q1 not reached; Q4: 22.6 months; *p* = 0.047; Figure 3a). No OS differences or patterns were associated with ASI in neither the NT nor the NT M0 group (Figure 3b). Upon further analysis, OS according to type of neoadjuvant therapy revealed the opposite association of ASI with OS in the FOL subgroup compared with the RES and GEM subgroups: median OS was significantly longer in Q4 (*n* = 7), the highest quartile of ASI values, than in Q1-3 of FOL patients at 38.4 vs. 22.0 months (95% CI: 0–80.1 and 14.7–29.3, respectively; *p* = 0.045; Figure 3c). In the GEM subgroup (*n* = 7), however, which was only divided into high vs. low values due to the small group size, it was again low ASI, which was significantly associated with longer OS at 35.0 months (95% CI: 22.8–47.2) vs. 18.8 (8.0–29.5; *p* = 0.025; Figure 3d). Both subgroup analyses lacked significant differences after exclusion of M1 patients due to small sample size, despite showing the same trends. In the COMB subgroup, no significant associations between ASI values and OS were observed.

### 3.3.2. α-SMA/Aniline Blue and Survival 

In RES and RES M0 patients, α-SMA values were not statistically associated with OS, but a trend for improved survival with lower quartiles was seen (Figure 3e). However, in the NT group, high α-SMA values (Q3 + Q4) were associated with improved OS of 38.4 (22.1–44.6) vs. 22.3 months for Q1 + Q2 (15.7–28.8; *p* = 0.011), as well as in the NT M0 group (39.4 (35.2–43.5) vs. 18.8 (8.7–28.8); *p* = 0.010; Figure 3f). This was rooted predominantly in an association of high α-SMA values with improved OS in the FOL subgroup, reaching significance in FOL M0 patients with improved OS for Q3 + Q4 at 28.4 (21.9–54.9) vs. Q1 + Q2 at 16.9 (6.9–26.8; *p* = 0.016; data not shown) (Figure 3f). In the COMB and GEM subgroups, no significant associations of α-SMA with OS were observed despite a similar trend.

In the analysis of AB stainings, Q4 (representing the highest collagen content) was associated with significantly longer OS than Q1 in the NT group (22.1 (16.0–28.2) vs. 35.0 (33.4–36.5), *p* = 0.042; data not shown). Despite a consistent trend for improved OS with higher AB values in the FOL and GEM patients, no further significant associations were found in NT and its subgroups. In the RES group, no trends nor associations of AB values with OS were observed.

### 3.3.3. Fibrogenic, Inert, Dormant and Fibrolytic Stroma

**Figure 4:**
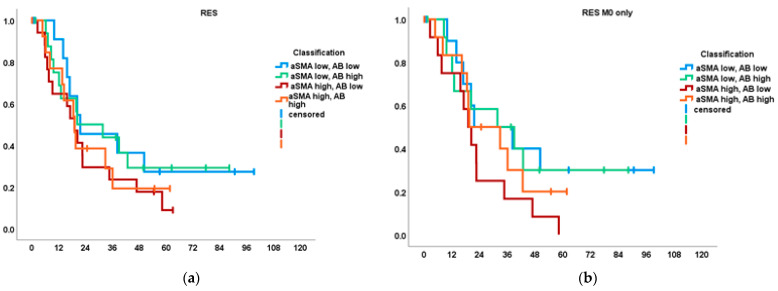

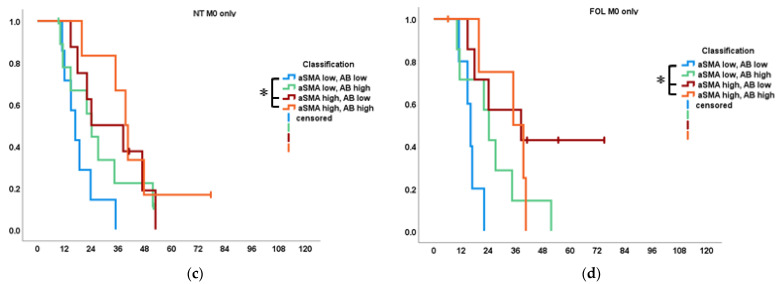
Overall survival (OS; months) of subgroups by classification of high and low expression of α-SMA and AB, respectively. (**a**) OS of RES; *n* = 14 low/low, 17 low/high, 17 high/low, 14 high/high; (**b**) OS of RES M0; *n* = 11, 13, 12, 14; (**c**) OS of NT M0; ✻ low/low vs. high/low *p* = 0.032; vs. high/high *p* = 0.002; (**d**) OS of FOL M0; *n* = 5, 7, 7, 5: low/low median OS 16.2 (13.3–19.0); high/low 38.4 (1.7–75.0; ✻ *p* = 0.009); high/high 35.9 (15.8–54.1; ✻ *p* = 0.017).

As an alternative to the arithmetic ASI, we analyzed survival classified by combinations of high and low α-SMA and AB according to median expression, as described before [28], and again found differing associations with OS between RES and NT groups: while median OS was comparable between the classes in RES at a range of 19.1–21.6 months (Figure 4a; RES M0: 19.1–31.7, Figure 4b) and low α-SMA classes represented a trend for best outcomes in RES (Figure 4a), in NT, high α-SMA was associated with a distinct survival advantage. The “inert” = low α-SMA/low AB class universally had the worst outcome in NT, NT M0 (Figure 4c), FOL and FOL M0 (Figure 4d). This was significant in NT for the low α-SMA/low AB class (*n* = 9) at 18.8 months (14.3–23.3) vs. both “fibrolytic” stroma = high/low at 24.1 (*n* = 12; 2.2–46.0; *p* = 0.032) and “fibrogenic” high/high at 40.5 (*n* = 10; 33.2–47.7; *p* = 0.002; data not shown). Congruent results were obtained in the NT M0 group with significantly shorter median OS in the low/low class at 16.9 (12.0–21.8; *n* = 7) vs. high/low at 24.1 (*n* = 8; 1.6–46.6; *p* = 0.026) and vs. high/high at 39.4 (*n* = 6; 32.7–46.0; *p* = 0.005). For further survival statistics cf. Figure 4. COMB and GEM subgroup analyses for this classification remained nonsignificant without any patterns or trends (data not shown).

## 4. Discussion

Low ASI, signifying low myCAF density and/or high stromal collagen deposition, has earlier been shown to be a positive prognostic factor in upfront resected PDAC patients, and the results in our control group largely confirm these findings [12]. However, after NT with FOLFIRINOX, the significance of ASI appeared to be overturned in our cohort, and high ASI values were associated with improved survival. This result could be traced back to a significant association of high α-SMA values and thus myCAF density with improved survival in the FOL subgroup of NT. Furthermore, patients with low expression of both α-SMA and collagen had by far the poorest outcome after FOLFIRINOX, but a trend for longer survival in the untreated group, as did the α-SMA low/collagen high class. All the while, α-SMA values showed the least variability over treatment subgroups and patient characteristics, with no significant differences found in any comparisons. This leads us to hypothesize that classification by abundance of myCAFs plus collagen might be a potential prognostic marker after NT and, especially, FOLFIRINOX treatment. High collagen, on the other hand, was by itself significantly associated with improved OS in the NT group, yet, in the GEM subgroup collagen was distinctly lower than in all others. The latter finding explains highly varying ASI values after differing types of NT, hence, according to our data, the ASI appears to have limited validity in individuals pretreated with current first-line regimens.

Interestingly, tissue parameters were not statistically different after NT compared to RES, and only the GEM subgroup revealed a distinct pattern with higher ASI rooted in lower collagen content but nondifferent α-SMA values. The small subgroup sizes, however, certainly need to be considered and represent one of the limitations of our study. Another relative limitation might be the lack of pretherapeutic tissue samples to investigate stromal marker changes over the course of neoadjuvant treatment, although our major findings pertain not to differential expression but instead differential association of tissue markers with prognosis between treatment cohorts. Other groups have reported controversial results on stromal markers after NT: Nakajima et al. found reduced collagen content for several collagen subtypes but not reduced α-SMA in 25 patients after NT; collagen reduction furthermore correlated with radiographic chemotherapy response [29]. However, the cohort was not stratified by type of NT and comprised seven different regimens, including S-1, which is currently not part of any treatment algorithms outside of Asia. Furthermore, the study lacks data on survival analysis. Xie et al. found higher α-SMA in samples after FOLFIRINOX (*n* = 11) than after gemcitabine-based therapy (*n* = 11) and in untreated samples but higher collagen in both NT groups compared with untreated. ASI was higher in FOLFIRINOX-treated patients than after gemcitabine-based therapy, but there was no correlation of ASI with survival [25]. Contrarily, in a study on 31 samples of PDAC after mostly gemcitabine-based neoadjuvant therapy and 13 after FOLFIRINOX, Mota-Reyes et al. found a significantly lower ASI after neoadjuvant treatment [26]; however, a stratification according to the type of NT was not provided, and the cohort contained no nab-paclitaxel-treated patients. None of these three studies employed identical staining methods, and none correlated primary tissue parameters with survival. Despite an analogous trend in our GEM subgroup, we could not statistically confirm the findings of Miyashita et al., who demonstrated decreased myCAF density after gemcitabine/nab-paclitaxel treatment in resected patient specimens [30]; possibly, this is a mere question of the small sample size of this subgroup in our cohort. Survival was not stratified according to α-SMA values in their study.

Collagen in PDAC appears to have ambiguous relevance for prognosis, which has been shown to correlate with abundance of its specific subtypes rather than overall content [31]. Additionally, the multifactorial dynamics of EMT are difficult to separate from the postulated role of collagen for PDAC proliferation and metastasization [32]. Nevertheless, there is no doubt as to the central part of collagen in the orchestration of PDAC biology and therapeutic resistance [33]. Paclitaxel is known to suppress fibrosis by modulation of hepatic stellate cells in the liver [34] and in peritoneal cells [35], mainly through the TGF-β signaling pathway. Stromal disruption in PDAC after GEM was described by Alvarez et al. with decreased collagen component as well as morphological changes appearing as disorganized collagen bundles [19]. This could explain the distinctive low collagen values we found in the GEM group. The association of higher collagen content with improved OS after NT in our cohort is in line with previous findings [13,14,36] and, considering the collagen decrease in the GEM subgroup, warrants more investigation in mechanisms of nab-paclitaxel response. Our data furthermore showed a possible association of collagen content with sex, pointing towards lower values in female patients in the NT group, FOL and GEM subgroups and the M1 cohort. As female sex was additionally significantly associated with more advanced T-stage and M1 stage in our cohort, and due to the small sample size in the subgroups, the true significance of sex regarding the analyzed tissue parameters cannot be inferred from our data. Nevertheless, the consistency of these significances and trends over different subgroups prompts further studies on gender differences in NT for PDAC given their potential association with the important outcome measures of response and survival in our study.

To our knowledge, the direct association of improved OS with greater myCAF abundance after current first-line NT regimens has not been previously reported in patient samples. As the complex dynamic interaction of CAFs in PDAC is still gradually being discovered [6,10,11,15,37] and encompasses a magnitude of markers, activation states, pro- and anti-immunogenic as well as pro- and antitumorigenic roles, it would be misguided to pose mechanistic hypotheses from our descriptive, exploratory study. Yet, our findings might challenge the postulate of acquired chemoresistance through increased stromal fibrosis and stiffness [38] as well as point towards the synergistic relevance of the myCAF population for NT with FOLFIRINOX.

Although no direct connection has neither been proposed nor investigated thus far, available data seem to suggest a link of NT with myCAF for the improved prognosis we found most likely via their mutual—or potentially interdependent—immunomodulatory mechanisms: On the one hand, myCAFs have been shown to confer improved prognosis by immune modulation in preclinical and translational models [15], and stromal myCAF depletion impaired response to gemcitabine but increased immunosuppressive Tregs and tumor growth [39]. On the other hand, FOLFIRINOX appears to alter immune cell profiles in PDAC [40,41], with a decrease in immunosuppressive Tregs itself. In breast cancer, however, a subset of myCAFs have shown association with upregulated Treg markers [42,43], thereby highlighting the ambiguous role myCAFs have in desmoplastic cancers. Potentially, the prognostic advantage we found for myCAF-high tumors only after NT might be a result of these synergistic anti-immunosuppressive effects. Nevertheless, the reality is likely more intricate, given the abundance of immune cells and their numerous associations revealed to date [44]. The improved OS with higher α-SMA as well as collagen values we found seems to support the latest studies showing the importance of collagen for myCAFs to adopt their tumor-suppressive role [13,14]; yet our untreated controls exhibited different associations. Possibly, the low α-SMA stroma might also contain more of a CAF subtype or stromal niche maintaining PDAC stemness [6,45], thereby conferring relative chemoresistance. Lastly, PDAC stroma has been shown to reflect the tumor’s genotype, and myCAF abundance might function as a surrogate marker for a more chemoresponsive PDAC subtype [31].

Many of the most comprehensive and distinguished recent publications on CAFs in PDAC did either not include investigations into response to NT or investigated chemotherapeutics with little relevance in current clinical standard treatments, leaving stromal interactions in modern NT regimens to be elucidated. Our data thus represent a stratified analysis of two of the most widely used stromal parameters for PDAC in a translational setting, correlating primary tissue markers with patient survival and adding real-world data to the numerous, and in part conflicting, recent reports on the significance of collagen and myCAFs in PDAC and NT.

## 5. Conclusions

Our study shows distinct properties of the myCAF and collagen compartment in PDAC tumor stroma after NT with current first-line agents FOLFIRINOX and gemcitabine/nab-paclitaxel and their differential association with survival compared with stroma without NT. These results underline the importance of further research into chemoresistance and response mechanisms on stromal level. Prognostic significance of ASI in resection of PDAC is not retained after NT, and future studies reporting ASI after NT should provide stratification and analysis of primary tissue markers for comparability of results.

## Figures and Tables

**Table 1:** Comparison of patient characteristics.

	Type of Neoadjuvant Therapy										
	RES		FOL		COMB		GEM		Total		
	*n*	%	*n*	%	*n*	%	*n*	%	*n*	%	*p*-Value
Overall	69	59.0	31	26.5	10	8.5	7	6.0	117		NA
Age (median (IQR))	64.7 (12.5)		59.8 (12.9)		59.2 (14.8) ^a^		69.6 (14.8) ^a^		62.4 (13.4)		0.039 ^a^
Sex											
male	35	50.7	17	54.8	8	80.0	3	42.9	63	53.8	ns
female	34	49.3	14	45.2	2	20.0	4	57.1	54	46.2	
ASA score											
1	1	1.4	0	0.0	0	0.0	0	0.0	1	0.9	ns
2	28 ^b^	40.6	22 ^b^	71.0	5	50.0	4	57.1	59	50.4	0.030 ^b^
3	40 ^c^	58.0	9 ^c^	29.0	5	50.0	3	42.9.9	57	48.7	0.045 ^c^
Neoadj. RCTx											
no					6	60.0			113	96.6	NA
yes					4	40.0			4	3.4	
Type of surgery											
DP	16	23.2	7	22.6	0	0.0	2	28.6	25	21.4	ns
TP	28	40.6	13	41.9.9	3	30.0	3	42.9.9	47	40.2	ns
PD	25	36.2	11	35.5	7	70.0	2	28.6	45	38.5	ns
Extended resection											
no	39 ^d^	56.5	9 ^d^	29.0	2	20.0	2	28.6	52	44.4	0.017 ^d^
yes	30 ^d^	43.5	22 ^d^	71.0	8	80.0	5	71.4	65	55.6	
R-status											
R0	15	21.7	6	19.4	2	20.0	3	42.9	26	22.2	ns
R1	54	78.3	24	77.4	7	70.0	4	57.1	89	76.1	ns
R2	0	0.0	1	3.2	1	10.0	0	0.0	2	1.7	ns
T-stage											
T1	6	8.7	3	9.7	0	0.0	1	14.3	10	8.5	ns
T2	44	63.8	17	54.8	6	60.0	4	57.1	71	60.7	ns
T3	19	27.5	7	22.6	3	30.0	2	28.6	31	26.5	ns
T4	0	0.0	4	12.9	1	10.0	0	0.0	5	4.3	ns
N-stage											
N0	29	42.0	11	35.5	5	50.0	3	42.9	48	41.0	ns
N1	24	34.8	11	35.5	4	40.0	2	28.6	41	35.0	ns
N2	16	23.2	9	29.0	1	10.0	2	28.6	28	23.9	ns
Grading											
G1	1	1.4									NA
G2	30	43.5									
G3	38	55.1									
Path. regression											
minor			15	78.9	3	37.5	1	33.3			ns
major			4	21.1	5	62.5	2	66.7			ns
M-stage											
M0	58	84.1	24	77.4	7	70.0	5	71.4	94	80.3	ns
M1	11	15.9	7	22.6	3	30.0	2	28.6	23	19.7	ns
Tumor stage (UICC 8th)											
I	22	31.9	7	18.9	1	10.0	1	12.5	22	31.9	ns
II	22	31.9	8	21.6	4	40.0	2	25.0	22	31.9	ns
III	15	21.7	12	32.4	2	20.0	3	37.5	15	21.7	ns
IV	10	14.5	10	27.0	3	30.0	2	25.0	10	14.5	ns

NA: not applicable; ns: not significant; DP: distal pancreatectomy; TP: total pancreatectomy; PD: pancreaticoduodenectomy. Superscript characters indicate significantly different subgroups for *p* < 0.05.

## Data Availability

The data presented in this study are available on request from the corresponding author. The data are not publicly available due to protection of patient privacy.
